# Rapid Fluorescence Quenching Detection of *Escherichia coli* Using Natural Silica-Based Nanoparticles

**DOI:** 10.3390/s21030881

**Published:** 2021-01-28

**Authors:** S. N. Aisyiyah Jenie, Yuni Kusumastuti, Fransiska S. H. Krismastuti, Yovilianda M. Untoro, Rizna T. Dewi, Linar Z. Udin, Nina Artanti

**Affiliations:** 1 Research Center for Chemistry, Indonesian Institute of Sciences—LIPI, Building 452, Kawasan Puspiptek, Tangerang Selatan, Banten 15314, Indonesia; fran008@lipi.go.id (F.S.H.K.); yoviliandamaulitivauntoro@gmail.com (Y.M.U.); rizn001@lipi.go.id (R.T.D.); lina004@lipi.go.id (L.Z.U.); nina001@lipi.go.id (N.A.); 2 Department of Chemical Engineering, Faculty of Engineering, Universitas Gadjah Mada, Jalan Grafika No. 2, Yogyakarta 55281, Indonesia

**Keywords:** *Escherichia coli*, fluorescence, natural silica, nanoparticles, Rhodamine B, biosensor

## Abstract

The development of fluorescent silica nanoparticles (SNP-RB) from natural amorphous silica and its performance as an *Escherichia coli* (*E. coli*) biosensor is described in this paper. SNP-RB was derived from silica recovered from geothermal installation precipitation and modified with the dye, Rhodamine B. The Fourier Infrared (FTIR) confirms the incorporation of Rhodamine B in the silica matrix. Transmission Electron Microscopy (TEM) micrographs show that the SNP-RB had an irregular structure with a particle diameter of about 20–30 nm. The maximum fluorescence spectrum of SNP-RB was recorded at 580 nm, which was further applied to observe the detection performance of the fluorescent nanoparticles towards *E. coli*. The sensing principle was based on the fluorescence-quenching mechanism of SNP-RB and this provided a wide linear *E. coli* concentration range of 10–10^5^ CFU/mL with a limit detection of 8 CFU/mL. A rapid response time was observed after only 15 min of incubation of SNP-RB with *E. coli*. The selectivity of the biosensor was demonstrated and showed that the SNP-RB only gave quenching response only to live *E. coli* bacteria. The use of SNP-RB as a sensing platform reduced the response time significantly compared to conventional 3-day bacterial assays, as well having excellent analytical performance in terms of sensitivity and selectivity.

## 1. Introduction

A large number of bacteria, identified as pathogenic, are associated with changing disease patterns. These pathogenic bacteria are responsible for the incidence or re-incidence of infectious diseases. Furthermore, the continuous development of antibiotic resistance among a variety of bacterial diseases is now becoming a major threat to public health in the world [1,2,3]. Pathogenic bacteria detection using conventional methods (i.e., culture-based assay, immunological assay, and polymerase chain reaction-based assay) is costly, labor-intensive, less sensitive and time consuming for a broad spectrum of pathogenic bacteria. The culture-based methods, for example, require 3 to 7 days for identification [3,4,5]. Therefore, alternative or advanced detection methods with enhanced sensitivity and selectivity yet having shorter detection time and reasonable prices become a substantial challenge.

Currently, biosensor technology offers its contribution to the advanced detection method of pathogenic bacteria. Biosensors could provide rapid, sensitive, and selective detection of the presence as well as the amount of bacteria in various environments. A biosensor is defined as a device that uses specific biochemical reactions for the detection of analytes such as isolated enzymes, immunosystems, tissues, organelles or whole cells, and further converts these reactions into electrical, thermal or optical signals [6]. Based on this definition, a biosensor consists of three main parts, namely a bioreceptor, a transducer, and a signal processing system [7,8,9]. The bioreceptors are biological molecular species or living biological systems utilizing biochemical mechanisms to recognize the targeted analyte. The bioreceptors commonly used in a biosensor system are nucleic acid/DNA, antibody/antigen, enzyme, protein, peptides and cells. The interaction between analyte and bioreceptor produces biochemical changes or signals. The transducer converts these biochemical signals into an electric signal which can be amplified and changed into readable signals by the signal processing system. The common transduction system in biosensors is optical (i.e., fluorescence, surface plasmon resonance, and chemiluminescence), electrochemical (i.e., amperometric, potentiometric, impedance and conductometric), piezoelectric (i.e., bulk wave and surface acoustic wave), and calorimetric [9].

Optical biosensors have potential to be developed as point-of-care (POC) diagnostics providing some advantages such as fast diagnostic results (shorter testing duration), insensitive to electromagnetic noise, on-spot detection, small amount sample volume and can be easily integrated with information processing system [3,10]. Hence, such a biosensor is promising for the detection of pathogenic bacteria [3,11,12]. The detection mechanism generally relies on monitoring the change in an optical signal, followed by a recognition event between a functionalized material and a pathogen [10]. Jokerst et al., (2012) developed a POC diagnostic system using a paper-based analytical device (µ-PAD) for the detection of *Escherichia coli* O157:H7, *Salmonella typhimurium*, and *Listeria monocytogenes* in food samples as a screening system. The µ-PAD containing a chromogenic substrate was prepared from a paper-based microspot assay created by use of wax printing on filter paper. Detection process occurs when an enzyme of the specific pathogen reacted with a chromogenic substance inducing color changes [13]. A different approach for *E. coli* detection was performed by Barreiros dos Santos et al., (2013) [14] using electrochemical impedance spectroscopy. The *E. coli* biosensing system gave a wide linear range of 3 × 10–3 × 10^4^ CFU/mL. 

The emergence of nanotechnology enables the design and engineering of new advanced materials at the molecular scale, thus having important impacts in various applications, including in biosensor technology. Recent developments in the fluorescence-based biosensor are focused on the exploitation of inorganic nanoplatforms combined with fluorophores or organic dyes for enhancing the detection performance [15,16]. These incorporations offer several advantages over conventional biosensors, such as sensitivity (i.e., low limit of detection), selectivity (i.e., minimizing false-positive signals), real-time analysis, label-free detection, small sample volume required, high-throughput screening, a high degree of stability as well as the possibility to be miniaturized [3,10]. The combination of nanostructures and fluorogenic substances for optical detection of pathogenic bacteria has been explored by scientists worldwide.

Miranda et al., (2011) developed the colorimetric detection of pathogens based on the conjugation system between the β-galactosidase (β-Gal) enzyme as an anionic enzyme to provide signal amplification and gold nanoparticles as a cationic nanoparticle to inhibit enzyme activity without denaturation. Chlorophenol red β-d-galactopyranoside (CPRG) was involved in the system as a chromogenic substrate to provide a color readout. Using this system, the bacteria can be detected and quantified at a concentration of 1 × 10^2^ bacteria/mL in solution and 1 × 10^4^ bacteria/mL in a field-friendly test strip format [17]. Xue et al., (2018) developed a fluorescent biosensor to selectively and rapidly detect *E. coli* O157:H7. The biosensor used the double-layer channel with the immune magnetic nanoparticles for specific separation and efficient concentration of the target bacteria and the immune quantum dots (QDs) with a portable optical system for quantitative detection of the bacteria [18]. Previous works have also applied to so-called porous silicon microcavity to enhance the fluorescence signal in detecting enzymes such as matrix metalloproteinase (MMP) and L-lactate dehydrogenase (LDH) which gave a response to concentrations as low as femtomolar [19,20].

Herein, we design an optical biosensor based on fluorescence silica nanoparticles for the detection of *E. coli* as the pathogenic bacteria model. The fluorescence silica nanoparticles (SNP-RB) are synthesized from silica nanoparticles modified with Rhodamine B as the fluorophore. Silica nanoparticles are one of nanostructure materials used as a building block of biosensor due to its advanced properties such as high surface area, ability to bind biomolecules covalently to the surface, biocompatibility, and biodegradability [21,22]. In this study, the silica precursors for the nanoparticles were obtained from silica scaling of geothermal power plants, as reported in our earlier study [23]. Rhodamine B, which has been well known as a pink fluorescent dye for detecting bacterial lipases [24], is applied as the fluorophore. Hence, the modification of silica nanoparticles using Rhodamine B is expected to sensitively and selectively detect the presence of *E. coli*. 

## 2. Materials and Methods

### 2.1. Material

Geothermal sludge, as the raw material, was collected from Geodipa Power Plant, Dieng, Central Java, Indonesia. Sodium hydroxide (NaOH), cetyltrimethylammonium bromide (CTAB), and analytical grade of hydrochloric acid (HCl) 37% were purchased from Merck Chemicals (Merck KGaA, Darmstadt, Germany). Rhodamine B was purchased from Sigma-Aldrich (St Louis, MO, USA). All chemicals were used without further purification. Deionized water was used for all experiments (pH 5, 91 and conductivity 14.9 μS at 25 °C). *E. coli* INACC-B5 bacterial was cultured in the Biochemical Laboratory, Research Centre for Chemistry, Indonesian Institute of Sciences (LIPI).

### 2.2. Preparation of Fluorescent Silica Nanoparticles

Fluorescent silica nanoparticles were prepared using the sol-gel method using silica obtained from geothermal sludge as the precursor. A total of 20 g wash silica geothermal was mixed with 800 mL of 1.5 N NaOH at 90 °C for 60 min under constant stirring to form sodium silicate (Na_2_SiO_3_). The mixture was then filtered through filter paper to separate the sodium silicate solution from the solids. As much as 0.05 g Rhodamine B was then added to a sodium silicate solution, stirred, and then the mixture was titrated with 2 N HCl to form the gel phase. The gel was subsequently immersed in 2 wt.% of CTAB in deionized water at room temperature. After 18 h, the gel was filtered using filter paper, neutralized with deionized water until pH 7, and then dried overnight in the oven at 100 °C. For further experiments, the obtained dried powder was denominated as SNP-RB samples.

### 2.3. Characterization of Fluorescent Silica Nanoparticles

The SNP-RB samples were characterized using surface area analysis using the Brunauer–Emmett–Teller (BET) method, Transmission Electron Microscopy (TEM) and Fourier Infrared (FTIR) Spectroscopy. Nitrogen adsorption-desorption isoterms were conducted in 77 K on Micrometritics Tristar II 3020 2.00 porosimeter to obtain the BET surface area. The FSNP was degassed at 110 °C and 10^−4^ Torr pressure. The sample for TEM measurement was prepared by taking a suitable amount of fluorescent nanoparticles and then dropped onto a porous carbon film on a copper grid and dried in a vacuum. The TEM images were obtained with TEM Tecnai G-20 S-Twin (FEI, USA) equipped with Tungsten cathode and an Eagle CCD camera. The FTIR spectra were recorded on a FTIR Prestige-21 (Shimadzu, Japan) in transmittance mode, at 16 cm^−1^ resolution over the range of 400–4500 cm^−1^ with an accumulating average of 10 scans. The software used to generate spectra was IR Solution (Shimadzu, Japan).

### 2.4. Fluorescence Spectrophotometer Measurements

The fluorescence intensity of the SNP-RB samples was optimized by comparing the fluorescence intensity emitted from a different concentration of Rhodamine B dye within the nanoparticles. The concentration of Rhodamine B dye was varied from 1.56 × 10^−4^ to 1 × 10^−2^ g/g and the synthesis process followed the procedure mentioned earlier in Section 2.2. A total of 10 g for each variation of the SNP-RB was dissolved in 10 mL of deionized water. The fluorescent silica nanoparticles were transferred into a 5 mL cuvette and then the fluorescence intensity was recorded over the range of 550–750 nm using a fluorescence spectrophotometer Cary Eclipse (Agilent, Singapore) at an excitation wavelength of 553 nm. Measurements were performed in triplicate.

### 2.5. E. coli Sensing Experiments Using SNP-RB

#### 2.5.1. Preparation of *E. coli* Bacterial Culture

*E. coli* InaCC-B5 bacterial culture was obtained from the Research Centre for Chemistry—LIPI. As much as 100 mL of nutrient agar was prepared by dissolving 2.3 g nutrient agar powder in 100 mL milliQ water. Nutrient agar was sterilized in an autoclave for 15 min at 121 °C and then allowed to settle. A single colony of *E. coli* was added to the sterilized agar plate and incubated for 24 h at 37 °C in a shaking incubator. The cultured *E. coli* was then diluted with sterilized water for further sensing experiments.

#### 2.5.2. Detection of *E. coli* Using SNP-RB

A total of 0.5 mL of fluorescent silica nanoparticles, SNP-RB solution was added into 4.50 mL of *E. coli* suspension in a beaker glass which was covered with aluminum foil to avoid exposure of light. The mixture was allowed to react for 15 min. The reaction mixture were transferred into a cuvette and the fluorescent intensity was measured. As a positive control, 0.5 mL of SNP-RB was added into 4.50 mL of nutrient agar in a beaker glass and treated as above-mentioned procedures. The fluorescent intensity of the reaction mixture and the positive control were observed over the range of 550–750 nm using fluorescence spectrophotometer at excitation wavelength of 553 nm.

#### 2.5.3. Response Time Measurements

Response time of *E. coli* detection using SNP-RB was determined by calculating the decrease of the maximum intensity (%*I_loss_*) of fluorescence emission before and after the addition of bacteria at different incubation times (5, 10, 15, 30 and 60 min). The detection of *E. coli* using SNP-RB were conducted as above mentioned procedure with the concentration of bacterial stock solution of 10^7^ CFU/mL. Measurements were conducted in triplicate.

#### 2.5.4. Sensitivity Measurements of SNP-RB for *E. coli* Detection

The sensitivity of the sensing platform was determined by calculating the decrease of the maximum intensity (%*I_loss_*) of fluorescence emission of the SNP-RB solution before and after the addition of bacteria with different concentrations ranging from 10–10^7^ CFU/mL at the optimum response time. The sensing experiments were also carried out as above mentioned procedure for each variation of bacterial concentration. Measurements were conducted in triplicate.

#### 2.5.5. Selectivity Measurements of SNP-RB for *E. coli* Detection

The selectivity test in this study was conducted by comparing %*I_loss_* of the SNP-RB samples when detecting *E. coli* to that when detecting dead *E. coli*, *Pseudomonas* sp., *Bacillus subtilis* (*B. subtilis*) and *Staphylococcus aureus* (*S. aureus*). All bacteria were cultured to the same concentration of 10^7^ CFU/mL. Dead *E. coli* was obtained by keeping a liquid culture of *E. coli* in a laminar flow with UV light for an hour. The sensing experiments were conducted as the same procedure as the above sensing procedure. Measurements were conducted in triplicate.

## 3. Results and Discussion

### 3.1. Characterization and Fluorescence Emission of SNP-RB

The detection platform used in this study is the fluorescent nanoparticles SNP-RB, which is obtained from the modification of geothermal silica precipitate with the fluorescent dye, Rhodamine B. The chemical and physical properties of the silica precursor has been reported in our earlier works [23,25]. We hypothesize that using other silica sources as the precursor will differ in regard to its chemical composition, surface chemistry as well as physical properties. These factors will further affect the synthesis and modification of the nanoparticles. The use of different silica sources will also affect in its further application as a detection platform, which has been reviewed extensively by Prabha et al., (2020) [26].

**Figure 1:**
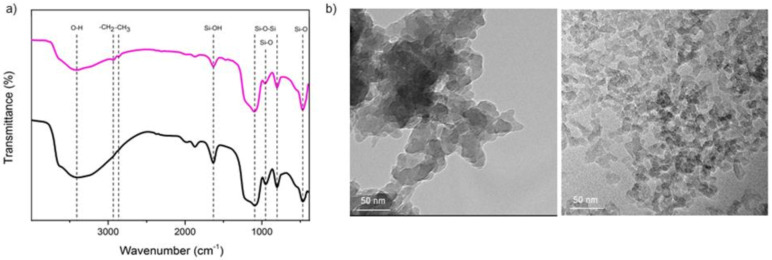
(**a**) Fourier Infrared (FTIR) spectra of silica nanoparticles (SNP) (black) and SNP-RB (pink) in transmittance mode; and (**b**) Representatives of Transmission Electron Microscopy (TEM) micrographs of the SNP (right) and SNP-RB (left) in 50 nm scale.

The sol-gel approach was chosen to develop the SNP-RB samples [27]. The chemical composition of the silica nanoparticles in the absence of the dye (SNP) and SNP-RB was observed by the FTIR spectra, as shown in Figure 1a. The SNP spectra showed prominent bands at 486 and 940 cm^−1^, which was attributed to the Si–O stretching vibrations indicating the presence of silica oxide in the nanoparticles. Peaks observed at 1093 and 802 cm^−1^ were assigned to the asymmetric and symmetric stretching vibrations, respectively, of the silica (Si–O–Si) network. A band around 1631 cm^−1^ was assigned to the bending vibration of water molecules bound to the silica lattice [28]. The broadband around 3400–3650 cm^−1^, which showed a maximum peak at 3410 cm^−1^, corresponds to the stretching vibration of –O–H bonds from the silanol groups and the remaining adsorbed water [29,30]. The SNP-RB spectra also show similar peaks to that of SNP; however, the intensity slightly decreased. The addition of the Rhodamine B dye was confirmed by the appearance of the peaks at 2864 and 2933 cm^−1^, which corresponds to the bending of –CH_2_ and –CH_3_ [28]. The TEM results showed that the SNP-RB has a homogenous range of size of 20–30 nm, as shown in Figure 1b.

**Figure 2:**
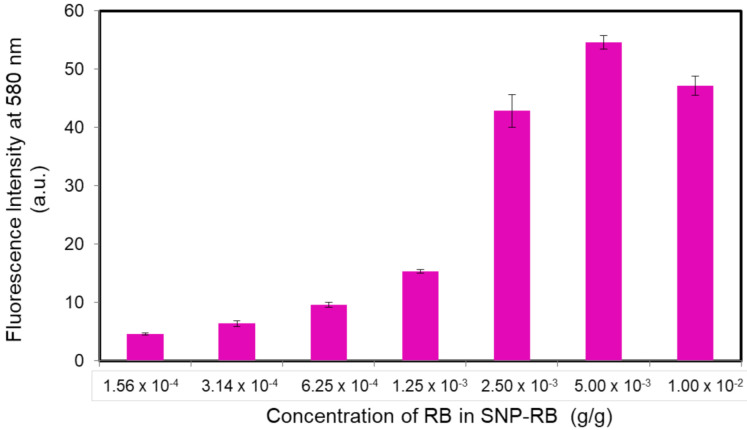
Correlation between maximum fluorescence intensity at 580 nm vs. Rhodamine B concentration in SNP-RB samples. The error bars were obtained after three separate experiments.

The SNP-RB was further observed for its fluorescence properties. Upon excitation at 553 nm, the fluorescence nanoparticle samples exhibited a broad emission peak with a maximum at 580 nm. The variation of Rhodamine B concentration in the nanoparticle samples from 1.56 × 10^−4^ to 1 × 10^−2^ g/g resulted in a change of the maximum intensity. Figure 2 shows that at dye concentrations from 1.56 × 10^−4^ to 1.25 × 10^−3^ g/g, the maximum intensity was relatively constant. However, when the concentration was further increased 2-fold to 2.5 × 10^−3^ g/g, the maximum intensity significantly increased to almost 3-fold and reached its optimum at 5 × 10^−3^ g/g of dye concentration. At higher Rhodamine B concentrations, the intensity slightly decreased due to the possible self-quenching of the dye molecules at saturated concentrations [31,32]. In the *E. coli* sensing experiments, we applied SNP-RB with a concentration rhodamine B of 2.5 × 10^−3^ g/g since it already exhibits high fluorescence intensity. The leaching test of SNP-RB in solution for 2 h was conducted to further confirm that the release of the dye was avoided and that the Rhodamine B dye was incorporated within the silica matrix (Figure S1, Supplementary Materials).

The use of organic dyes in conventional assays has several limitations, one of which is having low photostability and low fluorescence intensity. Hence, the dye must be in high concentration to attain the intended fluorescence intensity change. Following our previous studies, we have observed that the photostability and intensity of such dyes may be improved by incorporating the dyes with inorganic porous surfaces [15]. To achieve uniform size of the mesoporous structure for the nanoparticles, the sodium silicate gel was immersed in a CTAB solution and the surface morphology of SNP-RB was confirmed by surface area analysis (Table S1, Supplementary Materials). The SNP-RB contain large number of Rhodamine B dye in the silica matrix which further increased the fluorescence signal. Furthermore, when a fluorophore is in close proximity to nanostructure surface, the emission is drastically enhanced, whereas the fluorescence lifetime is decreased [15,33].

The fluorescence intensity of SNP-RB showed to have an enhancement of ~2-fold compared to the rhodamine B in its original state (Figure S2, Supplementary Materials). This was calculated from the ratio of the maximum fluorescence intensity of SNP-RB and RB which were 75.110 and 38.943 a.u., respectively, as shown in Table 1. The SNP-RB and RB were measured at the same concentration of 5 × 10^−5^ M in aquadest. The fluorescence enhancement of the fluorophore Rhodamine B incorporated in the silica nanoparticles or SNP-RB was corroborated by the fluorescence lifetime results of both SNP-RB and rhodamine B. The results in Table 1 showed a 0.9-fold decrease of SNP-RB lifetime compared to the fluorophore rhodamine B in its original state.

### 3.2. Analytical Performance of SNP-RB for the Fluorescence-Based Detection of E. coli

#### 3.2.1. Detection Mechanism

Rhodamine B is a xanthene dye commonly used in microbiology assays, pathology, and histology applications. Recent studies have reported the use of rhodamine dyes in the conventional methods of *E. coli* bioassays, known as pink assay [24,34]. In principle, the pink assay relies on the fluorescence intensity change of the dye. In the presence of *E. coli,* the fluorescence intensity of the rhodamine B dye will decrease, hence the detection of *E. coli* in samples. The work reported by Wang et al. stated the use of rhodamine-6G as a fluorescent label for the bacteria, due to the electrostatic interaction between the dye and the *E. coli* bacteria. In contrast, this resulted in increased fluorescence intensity of the labelled bacteria, hence the principle was applied for the detection of *E. coli*. 

Herein, we propose a detection mechanism for the detection of *E. coli* exploiting the fluorescence quenching of SNP-RB nanoparticles. The maximum fluorescence intensity change was used as the indication of sensing due to its sensitivity which has been reported to provide excellent limits of detection down to femto- and picomolar levels [19,35,36]. In fluorescence based sensing, the changes of fluorescence intensity is proportional to the excitation intensity, hence light emissions can be quantified down to a few photons using photomultiplier tubes or photodiodes and weak signals can be observed [15,37,38]. This resulted in a highly sensitive detection mechanism. In addition, the fluorescence based detection method provide excellent selectivity and specificity, suitable for the real-time detection of single or small molecules (i.e., molecular weight less than 1000 Da).

**Figure 3:**
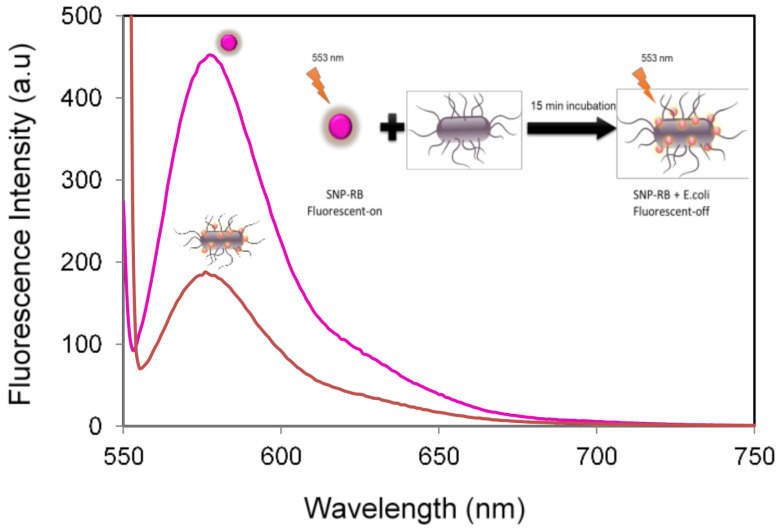
The fluorescence emission spectra of SNP-RB in deionized water (pink) and SNP-RB in the presence of *E. coli* proteins (orange). Concentration of SNP-RB and *E. coli* was 1 mg/mL and 1 × 10^7^ CFU/mL, respectively.

Samples SNP-RB were dispersed in sterilized water/aquadest and the maximum fluorescence intensity can be observed at 580 nm upon excitation at 553 nm, as shown in Figure 3 (pink spectra). Fluorescence quenching was later observed in the presence of *E. coli* bacteria, after 15 min of incubation (orange spectra). The fluorescence quenching was quantified as the percentage of intensity loss (%*I_loss_*), calculated using the following equation: *I_loss_* = (*I*_0_ − *I_t_*)/*I*_0_ × 100%, where *I*_0_ and *I_t_* is the maximum fluorescence intensity of SNP-RB before and after incubation with *E. coli*, respectively, after a certain period of incubation time, *t*. The %*I_loss_* after 15 min of incubation was calculated at 59.3%, indicating that the intensity was reduced to more than half compared to its initial state, *I*_0_. This result showed a more significant fluorescence change in the presence of *E. coli* compared to previous work using only rhodamine dye for the assay [24]. Hence, dyes incorporated into nanoparticles indeed increased the intensity difference after incubation with *E. coli*, leading to a more sensitive detection mechanism.

The absorption spectra of the SNP-RB sample upon *E. coli* aliquot addition corroborates the fluorescence-quenching mechanism. After detection, we observed the disappearance of the SNP-RB absorbance peak at 553 nm. The presence of *E. coli* indeed modified the scattering properties of the SNP-RB solution, limiting the fraction of excitation radiation, as shown in Figure S3 (Supplementary Materials).

This fluorescence-quenching principle is in agreement with previous studies for the detection of *E. coli* using rhodamine assays. Wang et al., (2016) have observed that after incubation, the dyes were attached to the *E. coli* walls. Such bacteria are known to have a high absorption capacity for fluorescent molecules due to the electrostatic force between the bacteria walls and the fluorescent molecules [34]. The *E. coli* walls are negatively charged, hence the attached dye will be protonated causing the reduced fluorescence intensity. This mechanism corroborates with other literatures on *E. coli* detection by fluorescence quenching [24,39]. In their study, the fluorescent dye was trapped inside the bacteria due to cell penetration. Subsequently, the fluorescence intensity is decreased after incubation, and the peptides inside *E. coli* were detected.

#### 3.2.2. Analytical Performance of SNP-RB as an *E. coli* Biosensor

**Figure 4:**
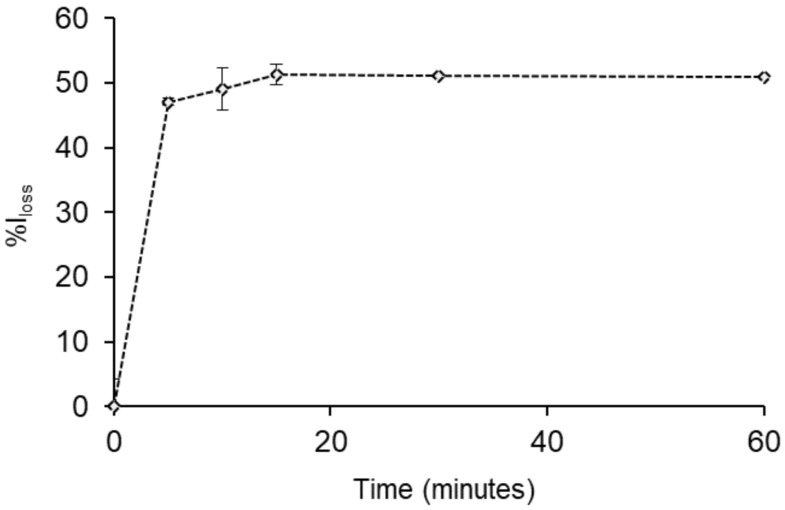
The detection time of *E. coli* (10^7^ CFU/mL) by 1 mg/mL of SNP-RB in solution plotted against the %*I_loss_*. The SNP-RB samples were excited at 553 nm and the %*I_loss_* were calculated from the maximum intensity at 580 nm.

We further examined the performance of the SNP-RB as a sensing platform for *E. coli* by performing fluorescence measurements at different incubation times. Figure 4 showed the %*I_loss_* plotted against different contact time between the SNP-RB and *E. coli*. The incubation was conducted in deionized water at room temperature, ensuring the constant concentration of the bacteria after being cultured. The plotlines clearly show that at 5 min the SNP-RB already gave a significant response, resulting in a fluorescence quenching of 47%. An increased incubation time to 60 min only gave a slight increase to constant %*I_loss_* percentage. At 15 min, the *I_loss_* remained constant at 51%, hence this incubation time was applied in our further experiments.

**Figure 5:**
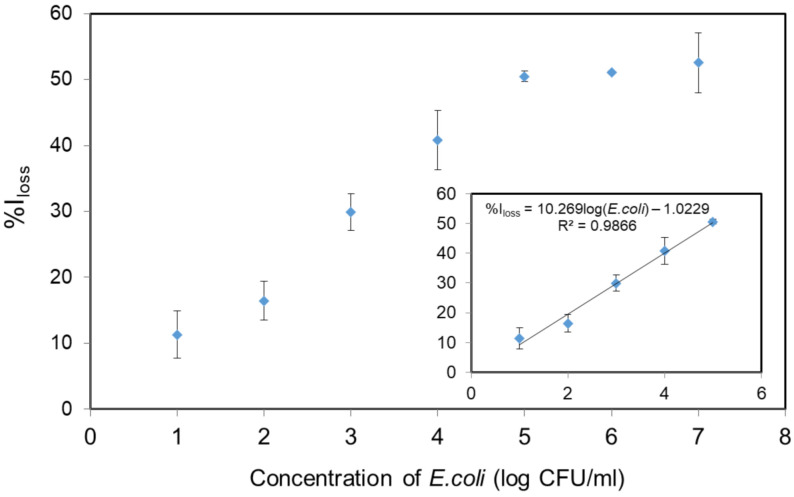
The correlation between %*I_loss_* of SNP-RB at 580 nm and *E. coli* concentrations after 15 min incubation. The inset shows the linear relationship over the range of 10–10^5^ CFU/mL.

To determine the sensitivity of the fluorescent nanoparticles for detection, the concentration of the bacteria was varied by diluting the *E. coli* in the nutrient agar. The detection of *E. coli* was performed by observing the decrease of fluorescence intensity (%*I_loss_*) of 1 mg/mL of SNP-RB solution at a constant incubation time of 15 min. The experiments were conducted in triplicate. The sensitivity performance was observed over the range of *E. coli* concentrations from 10 to 10^7^ CFU/mL and a linear correlation was found in the range of 10–10^5^ CFU/mL (%*I_loss_* = 10.269log (*E. coli*) − 1.0229; (*R*^2^ = 0.9866)) as shown in Figure 5. The limit of detection (LOD) was 8 CFU/mL, calculated using the equation of *y_b_* + 3*Std_b_*, whereas *y_b_* is the average fluorescence intensity loss measured for the blank control and *Std_b_* is the associated standard deviation [19,20]. This result is significantly lower than classic methods performed using an indirect ELISA technique which has an LOD of 10^3^ CFU/g of *E. coli* [40]. The LOD of the SNP-RB was found to be slightly higher compared to the detection of *E. coli* through the dyeing of bacteria with Rhodamine 6G of 2 CFU/mL [34]. The LOD results confirms the enhanced sensitivity of rhodamine B dye incorporated in silica nanoparticles for detecting *E. coli*. In addition, it is worth to note the simplicity of our detection system using SNP-RB, eliminating preparation and staining steps as found in conventional techniques.

**Figure 6:**
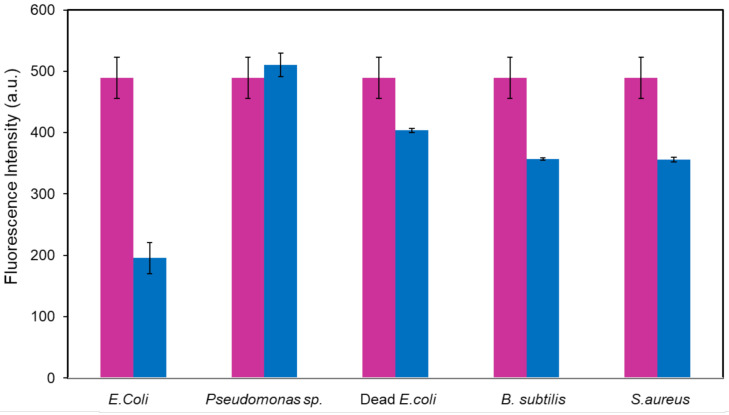
Comparison of fluorescence intensities of SNP-RB, before (pink) and after incubation with bacteria (blue). The error bars were obtained after conducting three separate experiments.

Having successfully demonstrated the detection of *E. coli* using SNP-RB via fluorescence quenching, the selectivity of the SNP-RB nanoparticles were further addressed. This was conducted by exposing it to another Gram-negative bacteria, *Pseudomonas* sp. and to dead *E. coli* bacteria, using the same procedure as above mentioned. The bacteria were adjusted to the same concentration of 10^7^ CFU/mL and incubation time was conducted for 15 min. Both results show that there was a small fluorescence intensity change of the SNP-RB. We have conducted the selectivity test compared to two Gram-positive bacteria, namely *B. subtilis* and *S. aureus*. The results showed that a decrease of fluorescence intensity was also observed in the presence of both Gram-positive bacteria, although not as significant to that of *E. coli*. The small fluorescence intensity change of 27% for both bacteria was comparable to that of dead *E. coli*, confirming the specificity of the fluorescence nanoparticles towards live *E. coli* (Figure 6). 

Overall, the detection of *E. coli* using fluorescent silica nanoparticles, SNP-RB, via fluorescence quenching was consistent with previous reported studies. Wang and co-workers, (2016) reported the assay using Rhodamine 6G labeled *E. coli* which required a culturing time of 12 h [34]. The group of Jokerst had reported a detection system which was able to detect bacteria until as low as 10 CFU/mL in a ready-to-eat meat sample and 12 h of enrichment time through color change [13]. A recent study has employed the use of quantum dots as the fluorophore for *E. coli* biosensor. The results showed that the biosensor was able to detect *E. coli* O157:H7 in spiked milk samples as low as 14 CFU/mL within 2 h detection time [18]. The use of nanocomposites comprised of paracetamol dimer and Au nanoparticles as *E. coli* detection platforms via fluorescence change has also been reported [41]. The nanocomposite platform was able to detect with a sensitivity of 10^2^ CFU/mL. The biosensor platform using SNP-RB resulted in much rapid response time and comparable analytical performances as summarized in Table 2. The *E. coli* biosensor based on SNP-RB showed a very low LOD of 8 CFU/mL with a wide linear range of 10–10^5^ CFU/mL. The biosensor also gave a very rapid response within 15 min of incubation. It is worth to note that the SNP-RB biosensor developed in this work is label free and does not require a long process of preparation stages.

## 4. Conclusions

The results provide a proof of concept of *E. coli* detection using SNP-RB nanoparticles via fluorescence quenching. To the best of our knowledge, this work reports for the first time the detection of microorganisms using a fluorescent nanoparticles based on natural silica from geothermal installation precipitate. The SNP-RB nanoparticles were obtained from the modification of silica geothermal as the precursor and Rhodamine B dye as the biorecognition molecule. FTIR, TEM and fluorescence spectroscopy were used to confirm the chemical, physical and optical properties of the SNP-RB samples, respectively. The fluorescence quenching after incubation of the SNP-RB samples with *E. coli* were observed upon excitation at 553 nm and maximum fluorescence emission of 580 nm. The SNP-RB samples provided excellent performance as *E. coli* biosensors giving rapid response time of 15 min with a wide linear range of 10–10^5^ CFU/ml and LOD of 8 CFU/ml. The biosensor also showed selectivity towards only live *E. coli* bacteria. The use of SNP-RB as a sensing platform reduced the response time compared to conventional bacterial assays as well having enhanced analytical performance. In addition, the biosensor is also cost-effective and environmentally friendly having been modified from natural silica precipitate. The developed SNP-RB biosensing platform serves as a proof of concept for future research related with the detection of other biomolecules via fluorescence and is expected to find applications in food, health and environmental industries.

## Figures and Tables

**Table 1:** The fluorescence maximum intensity (*I_max_*) and lifetime (*τ*) of SNP-RB and rhodamine B in H_2_O at concentration of 5 × 10^−5^ M. Excitation was conducted at 553 nm.

Compound	*I_max_*, a.u.	*τ*_H2O_, ms
SNP-RB	75.11 ± 3.35	0.009 ± 0.000
Rhodamine B	38.94 ± 9.04	0.01 ± 0.000

**Table 2:** Comparison of different methods for the detection of *E. coli* bacteria.

Method	Principle	LOD	Linear Range	Response Time	Comment	Refs
Fluorescence R6G-dyeing	Strong fluorescence was observed from *E. coli* stained with R6G dye.	2 CFU/mL	2–88 CFU/mL	12 h	Sensitive, simple, required 12 h of culturing bacteria	[34]
Colorimetric	Detection was observed via color change on a paper-based analytical device (*μ*PAD) which generated from the reaction of an enzyme from bacteria reacted with the chromogenic substance on the *μ*PAD.	10 CFU/mL	n.a.	12 h	Simple, required 12 h of bacteria enrichment	[13]
Fluorescence Quantum dots	The biosensor used a double channeled immune magnetic nanoparticles (MNPs) to separate the bacteria and quantum dots as the fluorescence reporter.	14 CFU/mL	8.9 × 10^0^–8.9 × 10^5^ CFU/mL	2 h	Sensitive, rapid	[18]
Fluorescence of nanocomposites	The detection of *E. coli* was based on the fluorescence quenching of paracetamol dimer and gold nanoparticle (AuNP) nanocomposites upon interaction with bacteria.	100 CFU/mL	10^2^–10^6^ CFU/mL	15 min	Simple, rapid	[41]
Electrochemical impedance spectroscopy (EIS)	The detection of *E. coli* was conducted via EIS on an antibody-modified gold electrodes on a self-assembled monolayer (SAM)	2 CFU/mL	3 × 10–3 × 10^4^ CFU/ml	45 min	Sensitive, rapid, label-free	[14]
Fluorescence of carbon quantum dots	The detection was based on the fluorescence quenching of carbon quantum dots-magnetic nanoparticles (CQDs-MNPs) labelled with aptamer and complimentary DNA, respectively.	487 CFU/mL	500–10^6^ CFU/mL	40 min	Simple, rapid	[42]
Fluorescence of upconversion nanoparticles	The detection was based on the fluorescence observed on the Yb-, Tm-, Fe-doped NaYF_4_ nanoparticles which are modified with polymyxin B in the prescence of *E. coli* bacteria.	36 CFU/mL	10^2^–10^7^ CFU/mL	2 h	Simple, sensitive	[43]
Fluorescence based on FRET	The detection of *E. coli* was based on the fluorescence quenching of fluorescence resonance energy transfer (FRET) between aptamer modified upconversion nanoparticles (UCNPs) as donors and layered tungsten disulfide (WS2) nanosheets as the acceptor.	17 CFU/mL	85–85 × 10^7^ CFU/mL	15 min	Simple, rapid, sensitive and selective	[44]
Immunofluorescence Assay	The detection of *E. coli* was conducted through fluorescence microscopy or fluorescence cytometry using fluorescein isothocyanate (FITC)-doped silica nanoparticles modified with *E. coli* antibody.	n.a.	n.a.	1 h	Rapid with intense luminescence and higher photostability	[45]
Fluorescence of SNP-RB	The detection was based the fluorescence quenching of SNP-RB in the presence of *E. coli* bacteria.	8 CFU/mL	10–10^5^ CFU/mL	15 min	Simple, rapid, sensitive, selective and label-free	This work

## Data Availability

Data sharing not applicable.

## Supplementary Materials

The following are available online at https://www.mdpi.com/1424-8220/21/3/881/s1, Figure S1: Fluorescence spectra of SNP-RB solution before (—) and after 2 h (---), Table S1: Specific surface area, pore size, pore volume and nanoparticle size of SNP and SNP-RB samples at reaction temperature of 90°C and aging time of 18 h, Figure S2: Fluoroscence spectra of the nanoparticles samples compared to the fluorophore, Rhodamine-B in H_2_O at the same concentration of 5 × 10^−5^ M. Figure S3: Absorbance spectra of PBS (blue spectrum), SNP-RB (red spectrum) and SNP-RB in the presence of *E. coli*.
