# Supplementary material for: Rapid Fluorescence Quenching Detection of Escherichia coli Using Natural Silica-Based Nanoparticles
Source: Sensors (Basel). 2021 Jan 28;21(3):881. doi: 10.3390/s21030881 (PMC7865786; doi:10.3390/s21030881)
Supplement: Supplementary file 1 [file sensors-21-00881-s001.pdf]

sensors

MDPI

Article

Rapid Fluorescence Quenching Detection of *Escherichia Coli* using Natural Silica-Based Nanoparticles

S. N. Aisyiyah Jenie [1,*], Yuni Kusumastuti [2,*], Fransiska S.H. Krismastuti [1], Yovilianda M. Untoro [1], Rizna T. Dewi [1], Linar Z. Udin [1] and Nina Artanti [1]

[1] Research Centre for Chemistry, Indonesian Institute of Sciences-LIPI, Building 452, Kawasan Puspiptek, Tangerang Selatan, Banten 15314, Indonesia fran008@lipi.go.id (F.S.H.K.); yoviliandamauliti-vauntoro@gmail.com (Y.M.U.); rizn001@lipi.go.id (R.T.D.); lina004@lipi.go.id (L.Z.U.); nina001@lipi.go.id (N.A.)

[2] Department of Chemical Engineering, Faculty of Engineering, Universitas Gadjah Mada, Jalan Grafika No. 2, Yogyakarta 55281, Indonesia

* Correspondence: siti045@lipi.go.id (S.N.A.J.); yuni_kusumastuti@ugm.ac.id (Y.K.); Tel.: +62-21-7560929 (S.N.A.J.); Tel.: +62-274-513665 (Y.K.)

Citation: Jenie, S.N.A..; Kusumastuti, Y.; Krismastuti, F.S.H.; Untoro, Y.M.; Dewi, R.T.; Udin, L.Z.; Artanti, N. Rapid Fluorescence Quenching Detection of *Escherichia Coli* using Natural Silica-Based Nanoparticles. *Sensors* **2021**, *21*, x. https://doi.org/10.3390/xxxxx

Academic Editors: Azhar Zam and Dedy H.B. Wicaksono

Received: 11 December 2020
Accepted: 25 January 2021
Published: 28 January 2021

Publisher's Note: MDPI stays neutral with regard to jurisdictional claims in published maps and institutional affiliations.

Copyright: © 2021 by the authors. Submitted for possible open access publication under the terms and conditions of the Creative Commons Attribution (CC BY) license (http://creativecommons.org/licenses/by/4.0/).

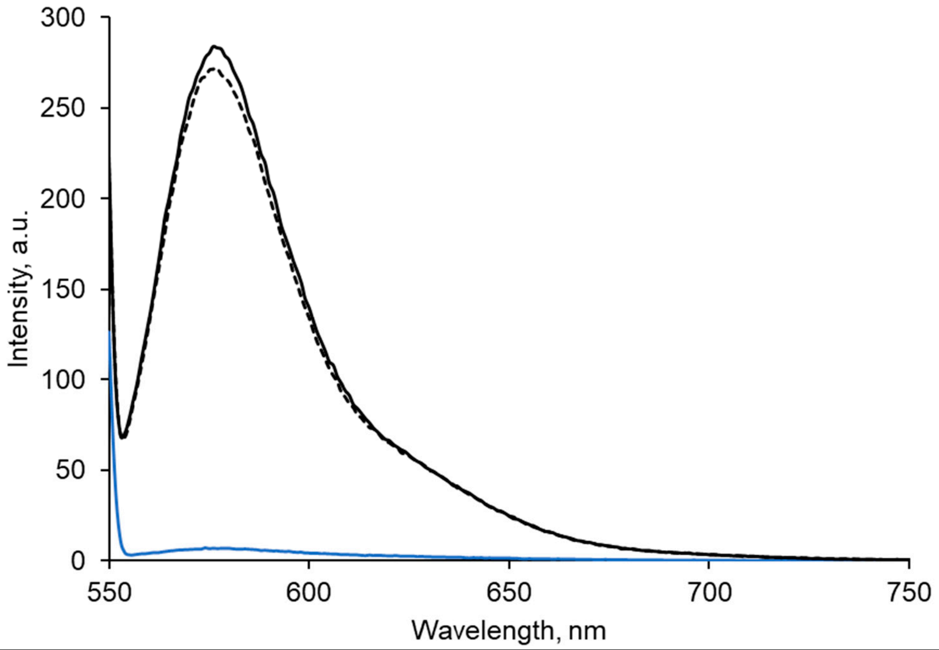

Figure S1. Fluorescence spectra of SNP-RB solution before (—) and after 2 hours (---). The fluorescence spectrum of aquadest (—) as the solvent was also measured.

Table S1. Specific surface area, pore size, pore volume and nanoparticle size of SNP and SNP-RB samples at reaction temperature of 90°C and aging time of 18 h.

No	Nanoparticles	Specification			
		Surface Area (m^2/g)	Pore Size (nm)	Pore Volume (cm^3/g)	Nanoparticle size (nm)
**1**	SNP	44.37	13.86	0.154	135.24
**2**	SNP-RB	190.23	27.89	1.326	31.54

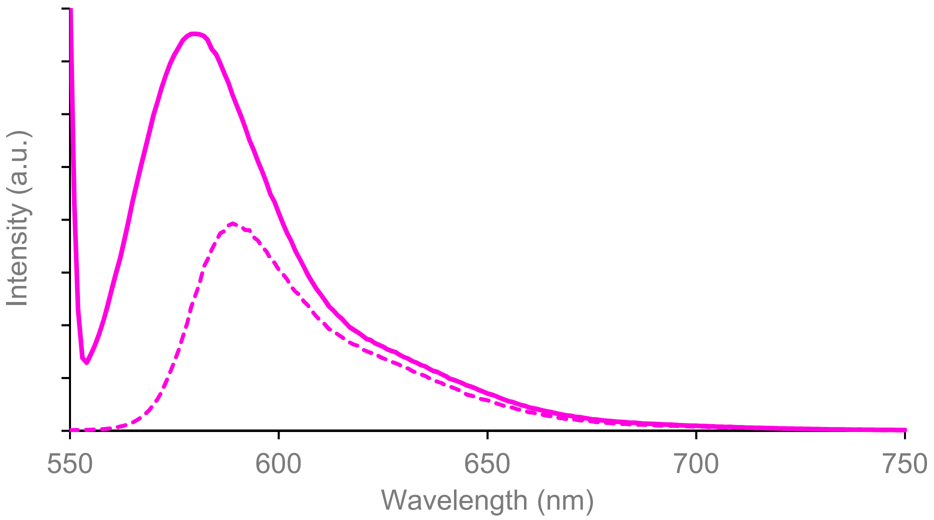

Figure S2. Fluoroscence spectra of the nanoparticles samples compared to the fluorophore, Rhodamine-B in H_2O at the same concentration of 5 x 10^{-5} M. The spectra corresponds to the emission of the fluorescent silica nanoparticles (FSNP) ( ) and Rhodamine-B (---).

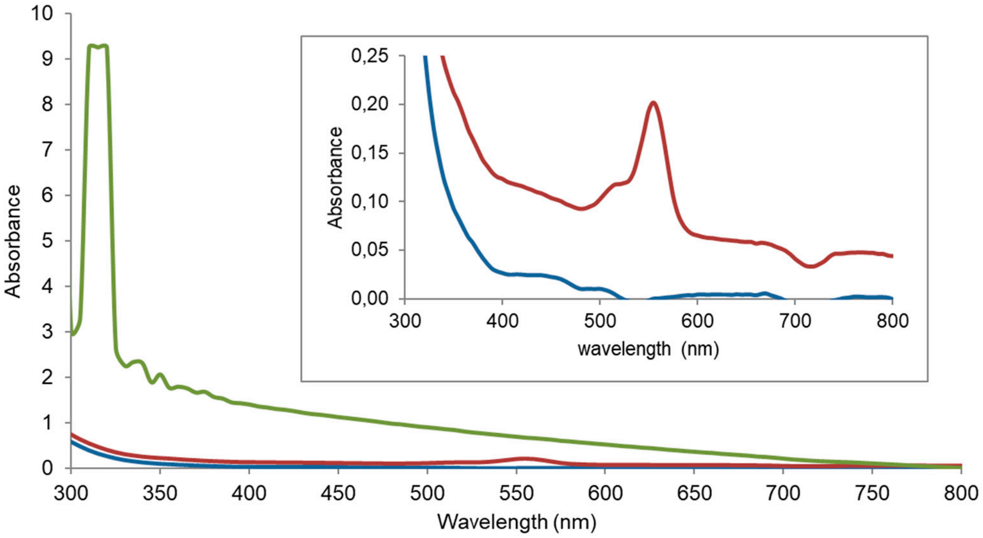

Figure S3. Absorbance spectra of PBS (blue spectrum), SNP-RB (red spectrum) and SNP-RB in the presence of *E.coli* proteins. Concentration of SNP-RB and E.coli was 1 mg/ml and 1 x 10^7 CFU/ml, respectively.

The concentration of SNP-RB solution after detection calculated using the Beer-Lambert equation was 3.56 mg/ml. This concentration value was higher than that before detection of 1 mg/mL, proving that the SNP-RB nanoparticles indeed interact with the bacteria.
